# Medical and Physical Intelligence

**Published:** 1806-12

**Authors:** 


### MEDICAL AND PHYSICAL INTELLIGENCE.

Dr. MATON, in a letter to Dr. BRADLEY, dated Spring Gardens, Nov. 7, 1806; writes as follows:

Dear Sir,

In compliance with your request, I send you, for insertion in the Medical and Physical Journal, an extract from a letter which I lately had the pleasure of receiving from Sir George Thomas Staunton, and which I agree with you in thinking will be acceptable to such of your readers as are not already acquainted with the facts contained in it.

"Canton, Feb. 20, 1806.

"It will not be uninteresting to you, to hear that we have at length introduced into China, Dr. Jenner's valuable discovery of the vaccine inoculation, and that, although the virus was obtained from Manilla by the assistance of the Spaniards, yet the surgeon of the British factory, Mr. Pearson, has the merit of pretty considerably derably diffusing, and I hope permanently establishing the practice, in this populous capital. It would have proved difficult to have so quickly overcome the objections and scruples of the Chinese against every kind of innovation, if Mr. Pearson had not hit upon the plan of writing a concise treatise on the discovery and mode of operation, which, by the assistance of a native Chinese, following the medical profession, I had the honour to translate into the language of this country. The work has been published and gratuitously distributed at the expence of the Company, and, as it is novel and unique in its kind, I take the liberty of inclosing you a copy."

The Chinese translation, which Sir George has done me the honour to present to me, is of the large octavo size, and consists of fourteen pages, on one of which are exhibited figures of the mature vaccine pustule; of the arm, with the proper place of puncture; the lancet charged with the matter; &c. It is well known that the Chinese surgeons are the most ignorant and unskilful of any perhaps throughout the civilized portions of the globe; hence too great pains could not be taken in regard to giving them directions sufficiently precise."

---

Mr. Dunning, one of the Surgeons of the Dock Jennerian Institution, has lately published a short pamphlet, entitled, Further Observations on the Practice of Vaccination, which concludes with the following deductions:

"I shall close this paper with some deductions, which are the result and ultimatum of my present opinions of the practice of vaccination; if true, they are not unimportant; they have the sanction of my medical friends to recommend them.

"1st. That looking at the practice of vaccination in all its effects and consequences, as it has hitherto presented itself to my observation, and *deliberately and severely judging it*, I subscribe implicitly at this time a belief, that the *few subjects* of incomplete vaccination, who are liable to a secondary impression of variolous poison, (and the number liable, seem to be very limited indeed) entirely possess all the exemptions and advantages attendant on favorable cases of the inoculated small-pox, exclusively of any risk of those afflicting and fatal events which so frequently follow and attach to the latter practice.

"2d. That if vaccination does not invariably assure to us full protection against the small-pox, exactly in the way we had at first expected; it has appeared however in *every instance I have yet seen*, to succeed completely in effecting that which ultimately amounts to the same important consequence; and hence, at this crisis, I deem it a duty to assert my unshaken conviction, that it is an invaluable blessing given by a kind Providence to mankind.

"3d. That it appears to me necessary the practice of vaccination should be considered under three characters; complete, incomplete, and spurious vaccination; that *complete vaccination*, there is every reason reason in the world to believe, *gives all that protection against small-pox*, which *can be derived from that inoculated disease;* that incomplete vaccination, there is the same reason to think, *generally* affords the *same protection*, *always* protects against danger, and in by far the greater number of the few subjects in this class, exposed to secondary impression, against even an approach to severe disease; and that spurious vaccination (to which the preceding observations have no reference) gives no protection at all.

"4th. That, how much soever the opinions I have here advanced, or have been elsewhere advanced by others on this subject, may be founded in truth and the nature of things, apparently, exceptions to them, from causes beyond the stretch of human power to detect, may now and then occur: that these, however, will not on a large scale, affect the general validity of them.

His utere mecum, si non rectius novisti.

---

Mr. OKES, of Cambridge, in a letter to the Editors, dated October 3, 1806, says,

"A gentleman who has retired from the profession of Surgery, has requested me to communicate to you the result of his practice in the ophthalmia of persons who have been in Egypt; there is nothing new in the remedy he made use of, but only in the manner of its application; and as it has frequently been successful, and as it has not, to the best of his recollection, been recommended by any author, or adopted by any practitioner, he trusts you will have no objection to give an account of it in your publication.

"Several soldiers who had been in Egypt, and had suffered there with the ophthalmia, have since their return to England been subject to returns of the inflammation, attended with most excruciating pain, very different from, and more violent than that which accompanies the common ophthalmia; for these patients he directed tinctura opii to be dropped into the eyes at bed-time, after the manner advised by Mr. Ware; great ease was obtained, but for the night only, the pain returning in the morning and continuing thro' the next day; at night, tinctura opii was again resorted to, and it again procured ease, but in the morning the pain returned with its former violence; he then prescribed tinctura opii to be dropped into the eyes in the morning, and to repeat the use of it every two or three hours in the course of the day, or as often as the pain returned; it was used many times in the day, and in two or three days the disease was most effectually subdued."

---

Professor KLAPROTH has taken great pains to investigate the component parts of native cinnabar; and he finds, as results of his experiments, that Japan cinnabar, exclusive of its foreign parts, contains

| | | | |
|---|---|---|---|
| Mercury | – | – | 84.50 |
| Sulphur | – | – | 14.75 |
| | | | 99.25 |

But

But that the cinnabar from Neumaerktel, in Carniola, consists of

| | | |
|---|---|---|
| Mercury | – – | 85.00 |
| Sulphur | – – | 14.25 |
| | | 99.25 |

Dr. Bozzini, of Frankfort, has invented an instrument, which he denominates the *light-spreader*. It is intended to afford an inspection of the interior of wounds, or the various parts of the human body, such as œsophagus, the vagina, the uterus, &c.—The inventor is preparing for the press drawings and descriptions of this curious instrument.

Dr. Clough will commence his Course of Lectures on the Theory and Practice of Midwifery, and the Diseases of Women and Children, at his House, No. 68, Berner's Street, Oxford Street, on Monday the 5th of January 1807, at the hour of ten in the morning, where particulars may be known. At the conclusion of the Course, Dr. C. will give a few Lectures on the Venereal Disease.

Mr. Alcock, No. 65, Well Street, has just commenced a Course of Lectures on Anatomy and Operative Surgery, conducted in a manner which insures to the Student, in a short space of time, a correct knowledge of those important branches.

Sir John Sinclair has nearly ready for publication, in four volumes octavo, his long promised Code of Health and Longevity; consisting of a detail of the circumstances which tend to promote health and longevity, with rules for preserving health.

